# Patients with volume-loaded right ventricle - quantification of left ventricular hemodynamic response to intervention measured by noninvasive pressure-volume loops

**DOI:** 10.3389/fphys.2023.1291119

**Published:** 2023-12-06

**Authors:** Pia Sjöberg, Sigurdur Stephensen, Håkan Arheden, Einar Heiberg, Marcus Carlsson

**Affiliations:** ^1^ Clinical Physiology, Department of Clinical Sciences Lund, Skåne University Hospital, Lund University, Lund, Sweden; ^2^ Department of Clinical Physiology, Skåne University Hospital, Lund, Sweden; ^3^ Wallenberg Centre for Molecular Medicine, Lund University, Lund, Sweden

**Keywords:** congenital heart disease, magnetic resonance imaging (MRI), atrial septal defect (ASD), tetralogy of fallot, arterial elastance (Ea), contractility

## Abstract

Volume loading of the right ventricle (RV) in patients with atrial septal defect (ASD) and patients with repaired Tetralogy of Fallot (rToF) affects the pumping mechanics of the left ventricle (LV). Intervention of the lesion will relieve the RV volume load however quantifiable impact on exercise capacity, arrhytmias or death are limited. A possible explanation could be remaining effects on the function of the LV. The aim of this study was therefore to investigate if hemodynamics of the LV differs between patients with RV volume load due to ASD or rToF and healthy controls and if they change after intervention. Eighteen patients with ASD, 17 patients with rToF and 16 healthy controls underwent cardiac magnetic resonance imaging (CMR) and maximal exercise test with continuous gas analysis. Reexamination was performed 13 ± 2 months after closure of the ASD in 13 of the patients and 10 ± 4 months after pulmonary valve replacement (PVR) in 9 of the patients with rToF. Non-invasive PV-loops from CMR and brachial pressures were analyzed. Stroke work (SW) and potential energy (PE) increased after ASD closure but not in ToF patients after valve repair. Patients with ASD or rToF had higher contractility and arterial elastance than controls. No major effects were seen in LV energetics or in peak VO_2_ after ASD closure or PVR. Peak VO_2_ correlated positively with SW and PE in patients with ASD (r = 0.54, *p* < 0.05; r = 0.61, *p* < 0.01) and controls (r = 0.72, *p* < 0.01; r = 0.53, *p* < 0.05) to approximately the same degree as peak VO_2_ and end-diastolic volume (EDV) or end-systolic volume (ESV). In ToF patients there was no correlation between PV loop parameters and peak VO_2_ even if correlation was found between peak VO_2_ and EDV or ESV. In conclusion, the LV seems to adapt its pumping according to anatomic circumstances without losing efficiency, however there are indications of persistent vascular dysfunction, expressed as high arterial elastance, which might have impact on exercise performance and prognosis. Future studies might elucidate if the duration of RV volume load and decreased LV filling have any impact on the ability of the vascular function to normalize after ASD closure or PVR.

## Introduction

Volume load of the right ventricle (RV) is a common feature in congenital heart disease, primarily in patients with atrial septal defect (ASD) and repaired Tetralogy of Fallot (rToF) with pulmonary regurgitation. In ASD patients there is a left-to-right shunt across the atrial septum and in rToF the pulmonary regurgitation leads to volume overload. The volume-loaded RV secondarily affects the pumping mechanics of the left ventricle (LV) (Broberg et al., 2011; Stephensen et al., 2014; Sjoberg et al., 2018a; Sjoberg et al., 2018b; Stephensen et al., 2018; Sjoberg et al., 2020). Interventional treatment of the underlying lesion, i.e., closing the ASD or repairing the pulmonary valve will in most cases result in a decrease in RV volumes but not all patients will experience increased exercise capacity and the impact on arrhythmias, or death is limited (Suchon et al., 2005; Geva et al., 2014; Ho et al., 2015; Bokma et al., 2018). A possible reason is the effect on the LV hemodynamics from a prolonged dilated RV. The knowledge in this field is limited. Pressure-volume (PV) loops are the “gold standard” for assessment of ventricular function (Suga and Shoukat, 1973), but they are rarely used in clinical routine due to the invasive procedure needed. Also, anatomical variations can make it difficult to correctly assess the ventricular volume in congenital heart disease. We have developed and validated a novel noninvasive technique for PV loops using cardiac magnetic resonance imaging (CMR) and brachial cuff pressure (Seemann et al., 2019; Sjoberg et al., 2021; Arvidsson et al., 2023). Using this non-invasive PV-loop algorithm we recently showed that the LV of patients with ASD and volume-loaded RV have normal ventricular efficiency in contrast to other patient groups with decreased cardiac output such as ischemic heart disease or dilated cardiomyopathy. The method has also been shown to be useful in assessing patients with rToF (Binka et al., 2022). The aim of this study was to determine if LV hemodynamics from PV loop analysis differs in patients with RV volume load due to ASD or rToF, if they change after ASD closure or pulmonary valve replacement (PVR), and if they are related to exercise capacity with the purpose to increase the understanding of heart failure in these patients.

## Material and methods

### Study design

Patients with rToF with a clinical referral for CMR were prospectively included as previously reported (Sjoberg et al., 2018a; Stephensen et al., 2019). Patients who had progressive right ventricular dilatation with end-diastolic volume >150 mL/m^2^, and/or symptoms and signs of heart failure (shortness of breath, fatigue, lightheadedness, chest discomfort or pain, high heart rate or arrythmia) underwent surgical PVR and were reexamined 10 ± 4 months postoperatively. Patients with pulmonary stenosis were not considered for the study. Patients with secundum ASD and Qp/Qs > 1.5 based on echocardiography or CMR were examined before transcutaneous ASD closure and 13 ± 2 months postoperatively. Healthy volunteers from a previously reported study (Stephensen et al., 2019) with normal ECG and blood pressure <140/90 mmHg, no cardiovascular medication, and no medical history of cardiovascular or other systemic disease acted as controls. All subjects had brachial cuff pressure measurements at rest and all subjects underwent a cardiopulmonary exercise test with continuous gas analysis for determination of exercise capacity. The study is performed in accordance with the tenets of the Helsinki Declaration (as revised in 2013) and has been approved by the authors’ National Review Board (Nr.: 213–436 and 2010–55).

### Cardiac magnetic resonance imaging

Cardiac magnetic resonance imaging was performed in the supine position with retrospective ECG gating using a 1.5 T Achieva (Philips Healthcare, Best, the Netherlands). Seven patients with rToF were examined using a 1.5 T Magnetom Aera (Siemens Healthcare, Erlangen, Germany). For assessment of ventricular volumes, a short-axis balanced steady-state free-precession (bSSFP) cine images covering the entire heart was acquired. Typical imaging parameters were: acquired temporal resolution 45 ms reconstructed to 30 time phases per heart beat; TE/TR 1.4/3 ms; flip angle 60°, slice thickness 8 mm with no gap. Two-dimensional free-breathing, through-plane phase-contrast (PC) flow measurements were performed in the ascending aorta and pulmonary artery to assess effective stroke volume, shunt and pulmonary regurgitation.

### Image analysis

**FIGURE 1:**
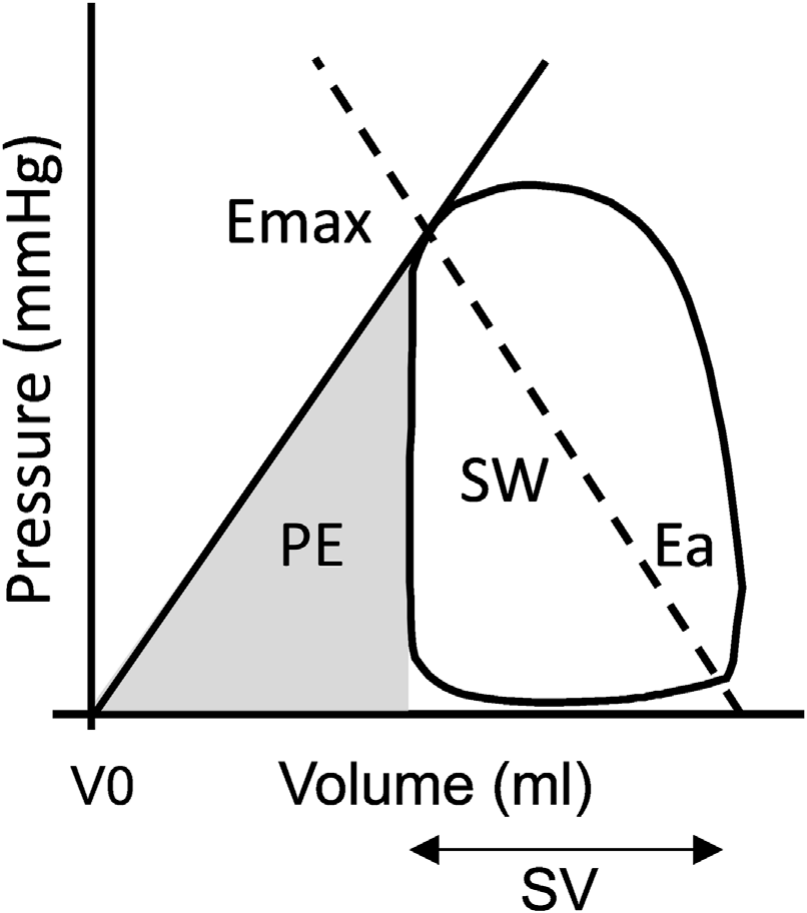
A: Schematic pressure-volume (PV) diagram. The PV loop area represents stroke work (SW), the energy produced by the left ventricle (LV) to eject the stroke volume (SV). Mean external power that the LV delivers is calculated as SW*(heart rate/60). Potential energy (PE) is obtained from the triangular area under the end-systolic PV relations curve and to the left of the PV loop. The energy consumption of a heart beat is thus SW + PE, which has been shown to be proportional to LV oxygen consumption [15]. The energy used per ejected volume is calculated as (SW + PE)/SV. SW as a fraction of total energy consumption, SW/(SW + PE), is a measure of ventricular energy efficiency (VE). Myocardial contractility (Emax) can be expressed as the slope of the relationship of end-systolic volume to end-systolic pressure. Arterial elastance (Ea) is the negative slope of the line from maximal ventricular elastance to the end-diastolic volume.

Left ventricular endocardial borders were delineated in all timeframes and analyzed using Segment software (http://segment.heiberg.se) with an in-house developed method for PV loop computation (Seemann et al., 2019; Sjoberg et al., 2021). The parameters derived from PV loops are explained in Figure 1.

### Haemodynamic variables

The hemodynamic variables that can be derived from PV-loop analysis are explained in detail in Figure 1. Stroke work is defined as the area within the PV loop and reflects the energy produced by the left ventricle to eject the stroke volume. The energy stored in the ventricle at the end of systole, and thus “wasted” energy that will be converted to heat, is the potential energy. Stroke work and potential energy adds up to the total energy consumption of a heart beat and has been shown to be proportional to oxygen consumption (Suga, 1979). The total energy consumption divided by the stroke volume shows how much energy is needed to eject a certain volume and stroke work as a fraction of total energy consumption is a measure of the ventricular efficiency. Contractility was defined as the slope between the point of maximal ventricular elastance and the volume at zero pressure, which was fixed to 0, (Sunagawa et al., 1985; Westerhof et al., 2010; Seemann et al., 2019; Sjoberg et al., 2021), and arterial elastance as the negative slope of the line between the point of maximal ventricular elastance on the PV loop and the end-diastolic volume.

### Cardiopulmonary exercise test

All participants performed a maximal exercise test with continuous gas analysis (Carefusion, Oxycon Pro, Jaeger, Würzburg, Germany) on a cycle ergometer (939E, Monark, Vansbro, Sweden). The patient performs the test with a face mask applied and the system analyzes oxygen uptake, carbon dioxide elimination, breathing frequency and tidal volumes, using breath by breath technique. The breathing gases are analyzed continuously during the test. The workload was increased until exhaustion, at which point peak oxygen uptake (VO_2_) was registered. The participants were encouraged to continue until the respiratory exchange ratio was 1.1 or more to ensure maximal exertion. Twelve-lead ECG was continuously recorded, and blood pressure was measured at rest and every minute during exercise. Peak VO_2_ was presented as milliliters per minute per kilogram, and as percentage of predicted value according to reference values for peak VO_2_ from the Study of Health in West Pomerania (SHIP) (Gläser 2010).

### Statistical analysis

GraphPad (La Jolla, CA; United States) was used for statistics. Continuous variables are presented as mean ± SD or median and interquartile range [IQR] and categorical variables as absolute numbers and percentages. Student’s t-test was used to evaluate differences between subjects. Paired t-tests were used to assess differences in patients before and after PVR or ASD closure. Results with a *p*-value below 0.05 were considered statistically significant.

## Results

Patients’ characteristics are shown in Table 1. Patients with ASD were older than patients with rToF and controls and had a higher proportion of women. Eight patients with ASD (44%) were on medication; 5 on betablockers (28%), 3 on diuretics (17%), one had ACE inhibitor (5%) and one was treated with Flecainide (5%). None of the patients with rToF had any medication due to their heart condition. Both patient groups had lower left ventricular end-diastolic volumes and stroke volumes, higher right ventricular volumes and lower peak oxygen uptake than controls. Patients with rToF had decreased right ventricular ejection fraction in contrast to patients with ASD who had normal ejection fraction.

**TABLE 1:** Characteristics of patients with atrial septal defect and patients with tetralogy of Fallot before transcatheter closure or pulmonary valve replacement and controls.

Mean ± SD or median [IQR]	ASD n = 18	rToF n = 17	Controls n = 16
Age (years)	47 [34–69] ** ###	24 [21–43]	32 [25–41]
Sex (men/women)	5/13	12/5	13/3
NYHA class	I: 8 (44%), II: 7 (39%), III: 3 (17%)	I: 13 (76%), II: 4 (24%)	I: 16 (100%)
Heart Rate (bpm)	73 ± 11	69 ± 12	65 ± 10
BSA (m^2^)	1.9 ± 0.2	1.9 ± 0.2	1.9 ± 0.2
SBP (mmHg)	124 ± 23	123 ± 18	122 ± 11
DBP (mmHg)	75 ± 16	78 ± 10	74 ± 10
LVEDV (mL)	139 ± 32 ****	158 ± 30 **	186 ± 28
LVESV (mL)	63 ± 19 **	73 ± 16	81 ± 17
LVSV (mL)	77 ± 18 ****	86 ± 22 **	104 ± 16
LVEF (%)	55 ± 7	54 ± 7	56 ± 5
CO (L/min)	5.5 ± 1.4 **	5.7 ± 1.0 **	7.0 ± 1.6
CI (L/min/m^2^)	2.9 ± 0.5 **	3.0 ± 0.4*	3.6 ± 0.8
RVEDV (mL)	300 ± 91 **	303 ± 72 ***	212 ± 44
RVESV (mL)	138 ± 51 **#	173 ± 46 ****	97 ± 28
RVSV (mL)	162 ± 51 **#	129 ± 35	115 ± 20
RVEF (%)	54 ± 7 ####	43 ± 6 ****	56 ± 6
Qp/Qs	1.9 ± 0.7	-	-
PR (%)	-	40 ± 9	-
Peak VO_2_ (mL/kg/min)	25 ± 6 ****	29 ± 9 ****	46 ± 8
Peak VO_2_ (% of predicted)	88 ± 19 ***	86 ± 18 ****	133 ± 21

ASD, atrial septal defect; rToF, repaired Tetralogy of Fallot; PVR, pulmonary valve replacement; NYHA, class, New York Heart Association Functional Classification; BSA, body surface area; HR, heart rate; SBP, systolic blood pressure; DBP, diastolic blood pressure; LVEDV, left ventricular end-diastolic volume; LVESV, left ventricular end-systolic volume; LVSV, left ventricular stroke volume; LVEF, left ventricular ejection fraction; CO, cardiac output; CI, cardiac index; RVEDV, right ventricular end-diastolic volume; RVESV, right ventricular end-systolic volume; RVSV, right ventricular stroke volume; RVEF; right ventricular ejection fraction; Qp/Qs, pulmonary to systemic flow ratio; PR, pulmonary regurgitation; VO_2_, oxygen uptake.

*p* < 0.05, **p < 0.01, ***p < 0.001, ****p < 0.0001 ASD, or rToF vs. controls.

*p* < 0.05, ##*p* < 0.01, ###*p* < 0.001, ####*p* < 0.0001 ASD, vs. rToF.

Table 2 shows the characteristics of patients who received ASD closure or PVR. Patients with ASD increased their left ventricular volumes and decreased their right ventricular volumes after closure of the defect. Patients with rToF had no change in left ventricular volumes after PVR, however right ventricular volumes decreased.

**TABLE 2:** Characteristics of patients with atrial septal defect and patients with tetralogy of Fallot before and after transcatheter closure or pulmonary valve replacement.

Mean ± SD or median [IQR]	ASD before closure *n* = 13	ASD after closure *n* = 13	rToF before PVR *n* = 9	rToF after PVR *n* = 9
Age (years)	45 [37–69]	46 [38–70]	38 ± 13	39 ± 13
Sex (men/women)	3/10	3/10	8/1	8/1
Heart Rate (bpm)	71 ± 10*	61 ± 10	70 ± 16	65 ± 15
BSA (m^2^)	1.9 ± 0.2	1.9 ± 0.2	2.1 ± 0.2	2.1 ± 0.2
SBP (mmHg)	120 ± 24	126 ± 17	135 ± 15	132 ± 11
DBP (mmHg)	71 ± 12	74 ± 7	84 ± 8	83 ± 9
LVEDV (mL)	143 ± 37 ***	159 ± 38	165 ± 33	172 ± 39
LVESV (mL)	66 ± 21*	72 ± 23	76 ± 15	75 ± 23
LVSV (mL)	78 ± 20 ***	87 ± 17	89 ± 28	97 ± 26
LVEF (%)	55 ± 6	56 ± 5	54 ± 9	56 ± 8
CO (L/min)	5.5 ± 1.3	5.4 ± 0.9	5.9 ± 1.0	6.0 ± 1.0
CI (L/min/m^2^)	2.9 ± 0.5	2.9 ± 0.4	3.0 ± 0.4	2.9 ± 0.4
RVEDV (mL)	291 ± 101 **	207 ± 46	343 ± 60 ***	242 ± 46
RVESV (mL)	133 ± 50	109 ± 25	195 ± 36*	147 ± 34
RVSV (mL)	158 ± 54 **	99 ± 25	147 ± 38 ***	95 ± 27
RVEF (%)	55 ± 6 ***	47 ± 6	43 ± 6	39 ± 7
Qp/Qs	1.8 ± 0.7	-	-	-
PR (%)	-	-	45 ± 5	-
Peak VO_2_ (mL/kg/min)	25 ± 5	28 ± 7 (*n* = 12)	25 ± 9	27 ± 8
Peak VO_2_ (% of predicted)	87 ± 21	94 ± 24 (*n* = 12)	81 ± 19	82 ± 14

ASD, atrial septal defect; rToF, repaired Tetralogy of Fallot; PVR, pulmonary valve replacement; BSA, body surface area; HR, heart rate; SBP, systolic blood pressure; DBP, diastolic blood pressure; LVEDV, left ventricular end-diastolic volume; LVESV, left ventricular end-systolic volume; LVSV, left ventricular stroke volume; LVEF, left ventricular ejection fraction; CO, cardiac output; CI, cardiac index; RVEDV, right ventricular end-diastolic volume; RVESV, right ventricular end-systolic volume; RVSV, right ventricular stroke volume; RVEF; right ventricular ejection fraction; Qp/Qs, pulmonary to systemic flow ratio; PR, pulmonary regurgitation; VO_2,_ oxygen uptake.

*p* < 0.05, **p < 0.01, ***p < 0.001 ASD, or rToF before vs. after closure or PVR.

**FIGURE 2:**
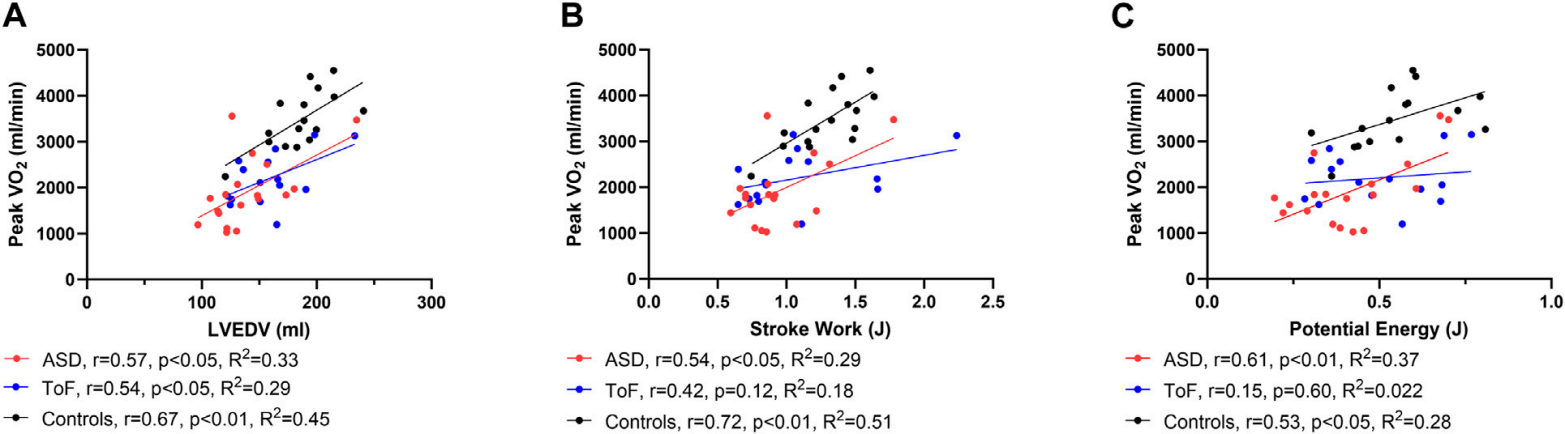
Scatter plot showing correlation between left ventricular end-diastolic volume (LVEDV) **(A)**, stroke work **(B)** and potential energy **(C)**, and peak oxygen uptake (VO_2_) in patients with atrial septal defect (ASD) and Tetralogy of Fallot (ToF) as well as controls.

Peak oxygen uptake did not change after ASD closure or PVR. Left ventricular end-diastolic and end-systolic volume correlated with peak VO_2_ in both patients with ASD and rToF before intervention, as well as in controls, Figure 2.

**FIGURE 3:**
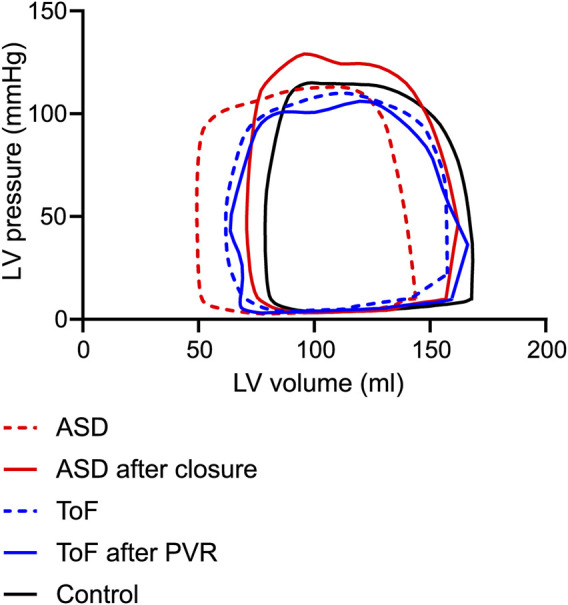
Example of PV loops from controls (black), patients with atrial septal defect (ASD) (red) and patients with repaired Tetralogy of Fallot (rToF) (blue). PV loops before ASD closure or pulmonary valve replacement (PVR) are shown with broken line and after with solid line.

Examples of PV loops for patients before and after intervention and controls are shown in Figure 3. The derived hemodynamic parameter values are shown in Tables 3, 4. Patients with ASD had lower stroke work (0.9 ± 0.3 J, *p* < 0.001) and potential energy (0.4 ± 0.2 J, *p* = 0.02) than controls (1.3 ± 0.3 J; 0.5 ± 0.1 J) before closure of the defect, and the values increased after closure (1.1 ± 0.3 J, *p* = 0.02; 0.5 ± 0.2 J, *p* = 0.04).

**TABLE 3:** Hemodynamic parameters in patients with atrial septal defect and patients with tetralogy of Fallot before transcatheter closure or pulmonary valve replacement and controls.

Mean ± SD	ASD *n* = 18	rToF *n* = 17	Controls *n* = 16
Stroke work (J)	0.9 ± 0.3 ***	1.1 ± 0.4	1.3 ± 0.3
Potential energy (J)	0.4 ± 0.2*	0.5 ± 0.2	0.5 ± 0.1
Stroke work + potential energy (J)	1.4 ± 0.4***	1.6 ± 0.5	1.8 ± 0.4
Ventricular efficiency (%)	69 ± 8	69 ± 7	70 ± 4
External power (J/s)	1.2 ± 0.4*	1.2 ± 0.4	1.5 ± 0.4
Contractility, Emax (mmHg/mL)	1.6 ± 0.6 **	1.4 ± 0.3 ***	1.1 ± 0.2
Arterial elastance, Ea (mmHg/mL)	1.5 ± 0.6 **	1.3 ± 0.3*	1.1 ± 0.2
Ventricular-arterial coupling, Ea/Emax	1.0 ± 0.3	1.0 ± 0.3	1.0 ± 0.2
Energy per ejected volume (J/mL)	18 ± 4	18 ± 3	17 ± 2

*p* < 0.05, **p < 0.01, ***p < 0.001 ASD, or rToF vs. controls.

**TABLE 4:** Hemodynamic parameters in patients with atrial septal defect and patients with tetralogy of Fallot before and after transcatheter closure or pulmonary valve replacement.

Mean ± SD	ASD before closure *n* = 13	ASD after closure *n* = 13	rToF before PVR *n* = 9	rToF after PVR *n* = 9
Stroke work (J)	0.9 ± 0.3*	1.1 ± 0.3	1.3 ± 0.5	1.3 ± 0.4
Potential energy (J)	0.4 ± 0.1*	0.5 ± 0.2	0.5 ± 0.1	0.5 ± 0.2
Stroke work + potential energy (J)	1.3 ± 0.4**	1.7 ± 0.5	1.8 ± 0.6	1.8 ± 0.4
Ventricular efficiency (%)	68 ± 6	69 ± 5	68 ± 9	73 ± 7
External power (J/s)	1.1 ± 0.4	1.1 ± 0.3	1.4 ± 0.3	1.4 ± 0.2
Contractility, Emax (mmHg/mL)	1.5 ± 0.7	1.4 ± 0.4	1.4 ± 0.3	1.5 ± 0.4
Arterial elastance, Ea (mmHg/mL)	1.4 ± 0.5	1.3 ± 0.3	1.4 ± 0.4	1.2 ± 0.2
Ventricular-arterial coupling, Ea/Emax	1.0 ± 0.3	1.0 ± 0.2	1.0 ± 0.3	0.9 ± 0.2
Energy per ejected volume (J/mL)	17 ± 4	18 ± 4	20 ± 2	18 ± 2

*p* < 0.05, **p < 0.01, ***p < 0.001 ASD, or rToF before vs. after operation.

Both patients with ASD and rToF had higher contractility (1.6 ± 0.6 mmHg/mL, *p* = 0.003; 1.4 ± 0.3 mmH/mL, *p* = 0.021) and arterial elastance (1.5 ± 0.6 mmHg/mL, *p* = 0.005; 1.3 ± 0.3 mmHg/mL, *p* = 0.031) before intervention than controls (1.1 ± 0.2 mmHg/mL; 1.1 ± 0.2 mmHg/mL). There was no change in contractility after volume unloading of the RV in either group (ASD: 1.4 ± 0.5 mmHg/mL, rToF: 1.5 ± 0.4). Also, arterial elastance remained unchanged in both patient groups (ASD: 1.3 ± 0.3 mmHg/mL, rToF: 1.2 ± 0.2 mmHg/mL).

There was no difference in energy per ejected volume between patients with ASD or rToF and controls, and no change was noted after ASD closure or PVR.

Left ventricular end-diastolic volume showed a positive correlation with SW, r of 0.56, 0.83 and 0.81 for ASD, rToF and controls respectively. The corresponding determination coefficients (*r* ^2^) were 0.32, 0.65 and 0.69.

Stroke work and potential energy correlated with peak VO_2_ in patients with ASD and in controls but not in patients with rToF, Figure 2. There were no correlations between ventricular efficiency, contractility, or energy per ejected volume and peak VO_2_ in neither patients nor controls.

## Discussion

We have shown how LV hemodynamics from pressure volume loops differ between patients with ASD and rTOF and how these parameters are affected by ASD-closure and PVR respectively. The main findings are that 1) patients with ASD as well as rToF patients had normal energy consumption per ejected volume both before and after relieving the volume load of the right ventricle 2) patients with ASD or rToF had increased contractility and arterial elastance, possibly due to increased sympathetic tone and these parameters remained increased 1 year after intervention indicating lasting vascular dysfunction 3) PV loop analysis showed poorer relationship with exercise capacity compared to standard LV volumes.

### Ventricular volumes

Closure of the ASD or PVR resulted in a decrease in right ventricular volumes, LV volumes increased after ASD closure but remained unchanged after PVR as earlier reported (Stephensen et al., 2019; Sjoberg et al., 2020). Patients with ASD also showed a decrease in LV ejection fraction, which was normal before intervention, in line with Umemoto et al. (2020). Patients with ASD had lower left ventricular end-diastolic volume, end-systolic volume, stroke volume and cardiac index than controls, because of the left-to-right shunt across the atrial septum causing low left ventricular filling. The larger left ventricular volumes after ASD closure did however not lead to an increased cardiac output, since heart rate decreased. These results are in line with other studies (Teo et al., 2008; Yoshiba et al., 2021). The reason for lower left ventricular end-diastolic volume and stroke volume in patients with rToF compared to controls might be explained by reduced transpulmonary flow caused by the pulmonary regurgitation leading to low left ventricular filling (Kopic et al., 2017; Ylitalo et al., 2018). The LV volumes were however within normal range and the control groups ventricular volumes were towards the upper limit, which might exaggerate the difference. The lack of change in LV volumes after PVR is consistent with Heng et al. (2017).

### Energy consumption

The lower energy consumption per heartbeat in patients with ASD is explained by the lower ventricular volumes and stroke volume since patients did not differ from controls in energy per ejected volume. The higher contractility and arterial elastance indicate that patients with ASD likely have an increased inotropic drive at rest compared to controls to preserve cardiac output. After ASD closure, the heart rate decreased, stroke volume increased as did left ventricular energy consumption per heartbeat indicating a lowered sympathetic drive. Energy per ejected volume was however unchanged, likely because of a lower heart rate after ASD closure compared to before as well as an unchanged systolic blood pressure.

Patients with rToF did not differ significantly in left ventricular energy consumption from controls and no change was seen after PVR. Although the number of patients with rToF who underwent PVR is limited the results indicate similar response to decreased filling of the LV as in patients with ASD.

Although the total energy consumption of the left ventricle correlates strongly with myocardial oxygen consumption in studies performed on excised heart *ex vivo* (Suga, 1979), there are more variability in this relation when assessing human hearts *in vivo* (Chirinos, 2013). Thus, this must be taken in consideration why it is difficult to draw any clear conclusions of how myocardial oxygen consumption change in the studied patient populations after intervention.

### Ventricular-arterial coupling

Contractility, as defined in PV loop analysis, is the slope between V_0_ and the point on the PV loop where the elastance is maximal (just before the end-systolic volume). The PV loops were displaced more to the left in both patients with ASD and patients with rToF than in controls. Thus, contractility was higher, possibly due to increased inotropy that compensates for low left ventricular filling in these patients. Earlier studies using cardiac catheterization have shown similar results (Senzaki et al., 2001).

The increased inotropy is likely also the reason for the higher arterial elastance, Ea. The ratio between Ea and Emax is used to assess ventricular-arterial coupling (VAC) and is potentially a prognostic marker (Ky et al., 2013; Godfrey et al., 2018). A ratio around or below 1 is thought to be most favorable for the energy expenditure of the heart (Chantler et al., 2008; Chirinos, 2013). The VAC in the patients with volume-loaded RV and, to various extent, underfilled LV in this study was normal. This suggests that the systolic function of the left ventricle is sufficient to compensate for the lower preload thereby upholding sufficient cardiac output, balanced by an increased afterload. This in contrast to patients with acquired heart failure with reduced ejection fraction, where the VAC is high and is prognostic for adverse clinical outcomes (Ky et al., 2013; Ikonomidis et al., 2019).

A speculation is that long lasting increased inotropic state to maintain cardiac output might have long term effects on the arterial endothelium predisposing these patients to cardiovascular disease. In this study we have no direct measure of sympathetic tone and the decrease in heart rate in ASD patients after ASD closure indicates that the sympathetic drive is lower after intervention. However, increased blood pressure, contractility and arterial elastance suggest that the sympathetic tone still remains increased 1 year after intervention. High sympathetic activity reduces arterial distensibility and affects the endothelial function so there is an imbalance between endothelium-derived vasocontriction and vasodilatation (Quarti-Trevano et al., 2021). Studies have shown that adults with rToF or ASD have diminished endothelial function (Scicchitano et al., 2019; Goeder et al., 2022). Patients after heart-transplantation also have increased arterial elastance which has been proposed to play a role in late graft failure (Latus et al., 2023) but the prognostic value has not to our knowledge been studied in patients with ASD or rToF. It is however shown that patients with non-complex CHD have an increased risk of cardiovascular disease, disproportionate to the burden of other known risk factors (Saha et al., 2019). Arterial distensibility and endothelial function is important to tissue perfusion and correlates with exercise performance (Buscemi et al., 2013). This might be a factor adding to why the patients did not improve their exercise capacity after intervention. Studies have shown that the endothelial function can improve after ASD closure (Scicchitano et al., 2019), aortic valve replacement in aortic stenosis patients (Takata et al., 2015) and cardiac resynchronization therapy in heart failure (Santini et al., 2013). Further studies are needed to elucidate if early intervention and thus shorter duration with high sympathetic tone might decrease the risk of cardiovascular disease.

### Cardiopulmonary exercise test

Peak VO_2_ in patients with ASD did not change 12 months after ASD closure in contrast to what has been reported by other groups (Yoshiba et al., 2021). This could be explained by peak VO_2_ before intervention already being within the normal range of what was expected in contrast to earlier reports that have shown mildly impaired peak oxygen uptake in patients with ASD (Kempny et al., 2012).

Patients with rToF had peak VO_2_ in the lower normal range expected for their age, however higher compared to other reports rToF (Kempny et al., 2012; Sabate Rotes et al., 2015). Peak VO_2_ did not increase after surgery, in line with earlier studies (Babu-Narayan et al., 2014).

Peak VO_2_ in patients with ASD or rToF correlated to a similar degree with left ventricular volumes as in controls but with lower oxygen uptake for the same ventricular size. After intervention with ASD closure or PVR, left ventricular volumes increased in ASD but not in ToF, and regardless of the differences in changes of volumes, peak oxygen uptake did not increase significantly in any group.

## Limitations

The method for PV loop estimation requires a non-restrictive pathway from the brachial artery to the LV. None of the participants in the study had any significant aortic stenosis, why this is not judged to affect the results. The volume at the pressure 0 mmHg in the LV is set to be 0 (V_0_), although it most likely should be a small positive value in a healthy LV. The method has however been validated and shown good agreement with *in vivo* measurements (Seemann et al., 2019). The V_0_ could possibly be higher in patients, but in patients with volume-loaded right ventricle it is reasonable to believe that the approximation is still valid. The ventricular end-diastolic pressure also needs to be estimated, but the validation of the method showed low influence on the derived parameters within a range of 0–15 mmHg, and therefore this will probably not affect the results in this study. Controls and patients were not matched for age and sex, and this is acknowledged as a limitation. However, blood pressure, which one could expect to be higher in older persons, was not higher in the ASD group compared to controls and will therefore not affect the PV loop parameters. Finally, since the number of patients is limited, statistical evaluation is difficult. However, the results are congruent with expected physiological response to right ventricular volume load.

## Conclusion

The LV seems to adapt its pumping according to the anatomic circumstances in ASD and rToF without losing efficiency. However, there are signs of persistent vascular dysfunction, expressed as high arterial elastance, which might have impact on exercise performance and prognosis after treatment. Future studies might elucidate if the duration of RV volume load and decreased LV filling have any impact on the ability of the vascular function do normalize after ASD closure or PVR.

## Scope statement

There is a need to be better at defining when and how to perform intervention in patients with congenital heart disease to improve outcome and wellbeing patients. A prerequisite is to increase the understanding of the pathophysiology. Symptoms in patients with volume-loaded right ventricles due to atrial septal defect or tetralogy of Fallot do not always improve after operation which might be explained by remaining effects on left ventricular function. We thus investigated the hemodynamic response to pulmonary valve replacement or ASD closure by means of non-invasive Pressure-volume loops with cardiac magnetic resonance imaging. We found that the left ventricle seems to adapt its pumping according to anatomic circumstances without losing efficiency, however there are indications of persistent vascular dysfunction, expressed as high arterial elastance, which might have impact on exercise performance and prognosis.

## Data Availability

The datasets presented in this article are not readily available because patient data cannot be made available due to data privacy concerns. Other data will be made available upon reasonable request. Requests to access the datasets should be directed to pia.sjoberg@med.lu.se.
